# BRENDA, the ELIXIR core data resource in 2021: new developments and updates

**DOI:** 10.1093/nar/gkaa1025

**Published:** 2020-11-19

**Authors:** Antje Chang, Lisa Jeske, Sandra Ulbrich, Julia Hofmann, Julia Koblitz, Ida Schomburg, Meina Neumann-Schaal, Dieter Jahn, Dietmar Schomburg

**Affiliations:** Technische Universität Braunschweig, Braunschweig Integrated Centre of Systems Biology (BRICS), Rebenring 56, 38106 Braunschweig, Germany; Technische Universität Braunschweig, Braunschweig Integrated Centre of Systems Biology (BRICS), Rebenring 56, 38106 Braunschweig, Germany; Technische Universität Braunschweig, Braunschweig Integrated Centre of Systems Biology (BRICS), Rebenring 56, 38106 Braunschweig, Germany; Technische Universität Braunschweig, Braunschweig Integrated Centre of Systems Biology (BRICS), Rebenring 56, 38106 Braunschweig, Germany; Leibniz Institute DSMZ-German Collection of Microorganisms and Cell Cultures, Inhoffenstrasse 7 B, 38124 Braunschweig, Germany; Technische Universität Braunschweig, Braunschweig Integrated Centre of Systems Biology (BRICS), Rebenring 56, 38106 Braunschweig, Germany; Leibniz Institute DSMZ-German Collection of Microorganisms and Cell Cultures, Inhoffenstrasse 7 B, 38124 Braunschweig, Germany; Technische Universität Braunschweig, Braunschweig Integrated Centre of Systems Biology (BRICS), Rebenring 56, 38106 Braunschweig, Germany; Technische Universität Braunschweig, Braunschweig Integrated Centre of Systems Biology (BRICS), Rebenring 56, 38106 Braunschweig, Germany

## Abstract

The BRENDA enzyme database (https://www.brenda-enzymes.org), established in 1987, has evolved into the main collection of functional enzyme and metabolism data. In 2018, BRENDA was selected as an ELIXIR Core Data Resource. BRENDA provides reliable data, continuous curation and updates of classified enzymes, and the integration of newly discovered enzymes. The main part contains >5 million data for ∼90 000 enzymes from ∼13 000 organisms, manually extracted from ∼157 000 primary literature references, combined with information of text and data mining, data integration, and prediction algorithms. Supplements comprise disease-related data, protein sequences, 3D structures, genome annotations, ligand information, taxonomic, bibliographic, and kinetic data. BRENDA offers an easy access to enzyme information from quick to advanced searches, text- and structured-based queries for enzyme-ligand interactions, word maps, and visualization of enzyme data. The BRENDA Pathway Maps are completely revised and updated for an enhanced interactive and intuitive usability. The new design of the Enzyme Summary Page provides an improved access to each individual enzyme. A new protein structure 3D viewer was integrated. The prediction of the intracellular localization of eukaryotic enzymes has been implemented. The new EnzymeDetector combines BRENDA enzyme annotations with protein and genome databases for the detection of eukaryotic and prokaryotic enzymes.

## INTRODUCTION

BRENDA, BRaunschweig ENzyme DAtabase (www.brenda-enzymes.org), established in 1987, is the most comprehensive and widely used enzyme information resource worldwide. Initially, the enzyme data in BRENDA were published as a series of books (Handbook of Enzymes (1)). In 1998, the information collection was transformed into a publicly available database with a simple query system. With the fast-growing number of published data and improved knowledge in the ‘OMICS’ world the continuous integration of new high-quality data and new developments in BRENDA is essential to meet the requirements of the users in the scientific community in the fields of systems biology, biotechnology, medical and pharmaceutical research.

The main goal of BRENDA is to offer scientists of all areas within the life sciences access to full information on classified enzymes, their function, occurrence, genetic context, metabolic role, structure, and specificity. Additional tools for exploring pathways, linking metabolome data to biochemical pathways or for exploring the functional properties of enzyme-like active sites and substrate-enzyme interactions are provided.

Enzymes play an essential role in all processes of life, including metabolism, gene expression, cell division, the immune system, and others. Their functions, being also connected to most diseases or stress control, render them interesting targets for research and applications in biotechnology, medical treatments or diagnosis.

The enzyme information is classified with respect to the EC nomenclature, the enzyme classification system of the IUBMB (2). The hierarchical structure is displayed in the ‘EC Explorer’ in BRENDA. The enzyme classes are defined according to the type of catalyzed reactions, currently ∼7600 different reactions. The main core of BRENDA contains more than 5 million data, manually evaluated and extracted from ∼157 000 primary literature references, comprising information on ∼90 000 enzymes from ∼13 000 organisms, supplemented with information from text and data mining methods, data integration from external sources, and prediction algorithms. The results of the imported and processed data comprise disease-related data, protein sequences, 3D structures, predicted enzyme locations and genome annotations.

The manually extracted data in BRENDA are extended by information retrieved by text mining of PubMed literature abstracts (3), containing 1.9 million entries from 3.9 million references including information on enzyme-diseases, organism-tissues, cellular and subcellular localization, and kinetic data stored in the accessory repositories: DRENDA, FRENDA, AMENDA and KENDA (4–6).

A fundamental part is the BRENDA ligand database. The term ‘ligands’, covers all compounds interacting with enzymes, such as substrates and products, inhibitors, cofactors, activating molecules, etc., comprising macromolecules and ∼207 000 small molecules, which are stored as structures with their names and synonyms.

BRENDA offers an easy access to enzyme information. The user can perform text-based queries covering quick searches, full text or advanced searches. Structure-based queries for enzyme ligands are provided by drawing a chemical structure in the molecule editor for substructure, isomer and similarity searches. The visualization of enzyme data and information are presented in the recently completely revised BRENDA pathway maps, word maps and the 3D view of enzymes.

Additionally, BRENDA provides prediction tools, genome and taxonomy information, and the access to diverse enzyme-related ontologies, such as the BRENDA Tissue Ontology (BTO, (7)). The BTO, started in 2003, is a comprehensive structured encyclopedia of tissue terms and cell lines, including synonyms and definitions, from multicellular organisms. It provides direct access to the information of enzymes isolated from specific body parts, organs, tissues, cell types, and cell lines. The integration and comparability of the BRENDA Tissue Ontology (BTO) with other major ontologies like the gene ontology and others has been facilitated, which should lead to an even wider usage of the BTO in the future. A GitHub Repository has been created for the BTO (https://github.com/BRENDA-Enzymes/BTO/) and the ontology development kit used by nearly all major ontologies is now used to prepare upcoming versions of the BTO (ODK, https://douroucouli.wordpress.com/2018/08/06/new-version-of-ontology-development-kit-now-with-docker-support/).

Since 2015 the human anatomy atlas CAVEman, based on the ‘Terminologia Anatomica’, is linked to BRENDA to connect human enzymes to the detailed ontology of body parts, organs, and tissues (8,9). The subcellular localization of BRENDA enzymes is linked to the cellular component branch of the Gene Ontology (10). The organisms expressing the enzymes in BRENDA are linked to the ‘TaxTree Explorer’ which is based on the NCBI Taxonomy database (11). The ‘Genome Explorer’ displays and compares enzymes of a complete genome to allow the user to visualize the genomic environment of the gene of interest in different organisms. CATH and SCOPe cover the 3D structural classification of enzymes (12,13).

BRENDA offers two types of word maps giving a quick overview on enzyme research areas visualizing enzyme-specific terms (enzyme word maps) and organism word maps, focusing on organism-specific terms occurring in PubMed titles and abstracts. The BKMS-react module in BRENDA is a way to provide combined enzyme-catalyzed reactions of the four main metabolic databases BRENDA, KEGG, MetaCyc and SABIO-RK (14–16). In addition to the manually extracted information BRENDA integrates data of further external databases: IUBMB ExplorEnz, PubMed, UniProt, PDB, BacDive, NCBI-MeSH (17–20) and provides links to other databases (i.e. PROSITE, InterPro). Since NCBI Taxonomy identifiers are now available in BacDive the number of links from BRENDA to BacDive from organism related pages has been increased by orders of magnitude.

In 2018, BRENDA was appointed ELIXIR Core Data Resource (https://elixir-europe.org/platforms/data/core-data-resources) due to the fundamental importance to biological and biomedical research and long-term preservation of biological data (21). BRENDA is part of the de.NBI (https://www.denbi.de/, German Network for Bioinformatics Infrastructure), the German Node of ELIXIR. BRENDA belongs to the de.NBI service center for Biological Data (BioData) providing a wide variety of bioinformatic services and resources for life science-related research in academia and industry. Following the guidelines of ELIXIR (22) BRENDA established an international independent Scientific Advisory Board (SAB) allowing community input and providing permanent oversight. Furthermore, BRENDA supports ‘Open Science’ by providing its data within the scope of Creative Commons licenses (CC BY 4.0).

Since the last publication in 2019 new features and developments are implemented in BRENDA. The BRENDA pathway maps have been substantially revised and updated. The access of enzyme and metabolite information within the pathways has been improved for a more interactive and intuitive usability. The Enzyme Summary Page was redesigned for a quick and clear overview and an easy access to each individual enzyme. The visualization of the 3D protein structures is now presented with a new integrated NGL viewer. The new localization prediction of enzymes is now extended with eukaryotic enzymes. The EnzymeDetector for the prediction of enzymatic functions based on genome annotation was introduced in 2011. It is now available in a revised and updated form, including the extension to eukaryotic genomes. Further new developments focus on the optimization and improvement of the central working and management databases as well as the optimization of the automatic website check for testing hundreds of search combinations in BRENDA. Training and tutorial materials/videos are updated in terms of the new developments and updates.

## BRENDA CONTENT

The main core of BRENDA is composed of manually extracted literature data. The references provide data for 8083 enzyme classes (EC numbers). These classes also include transferred and deleted entries showing the ‘history’ of their classification. Some designated as ‘preliminary BRENDA-supplied EC number’ are also included. These are entries awaiting approval by the IUBMB. The number of EC classes has increased by 493, including 312 newly classified and 181 transferred and preliminary EC numbers since the last publication in 2019. The manual annotation procedure covers ca. 60 data and information fields, such as kinetic and stability data, catalyzed reactions, occurrence, protein-ligand interaction, experimental conditions, procedures for purification, crystallization, and cloning. Each value is connected to the organism of origin, a literature reference, and where available, the UniProt protein sequence ID. The characterized enzymes cover all taxonomic groups ranging from eukaryotes, archaea, bacteria to viruses. Since 2019 ∼11 000 new references have been manually annotated, and 4139 EC classes were updated. Table 1 shows the development of data content for a selection of data fields from 2016 to 2020 (23).

**Table 1.** Number of entries in selected data fields

Enzyme information	Entries 2016	Entries 2018	Entries 2020
Substrates and products	407 446	435 289	471 088
Inhibitors	196 548	207 441	226 424
Cofactors	14 382	15 964	16 954
Metals and ions	36 711	38 548	41 985
Activating compounds	26 761	27 653	29 025
*K*_M_-values	135 603	145 215	154 081
*K*_i-_values	38 378	39 927	42 468
*k*_cat_-values	62 445	68 963	75 441
Specific activity	45 773	48 001	50 238
IC_50_-values	49 842	54 230	60 923
Localization and source tissue	96 889	102 758	110 499
Enzyme names and synonyms	102 394	111 488	123 077
Citations (manually annotated)	146 221	155 422	168 008
Isolation and preparation/crystallization	88 849	96 765	107 365
Enzyme structure	158 397	196 071	286 714
Mutant enzymes	76 451	83 355	93 590
Enzyme stability	47 281	49 271	52 926
Enzyme application	15 080	16 441	18 798

The numbers refer to the combination of enzyme protein and literature reference. The term ‘enzyme protein’ refers either to a protein sequence or to a protein isolated from a given organism without its sequence having been determined.

## NEW DEVELOPMENTS AND MAJOR IMPROVEMENTS

### Enzyme summary page

The BRENDA Enzyme Summary Pages providing a fast and easy access to all available information of all enzymes are the most frequently called pages in BRENDA. All query strategies in BRENDA lead to these pages sooner or later. Additionally, users who look up enzyme information via a web search (i.e. Google) are often led to the BRENDA Enzyme Summary Pages. The information on these pages is growing quickly with each BRENDA update and the inclusion of new functionalities like the prediction of localizations. Therefore, the Enzyme Summary Pages have now been extensively revised and optimized for an improved usability: compact and clear first view, improved overview, easy navigation, individual selection of categories and data fields, and accelerated loading time to access the data.

**Figure 1.**
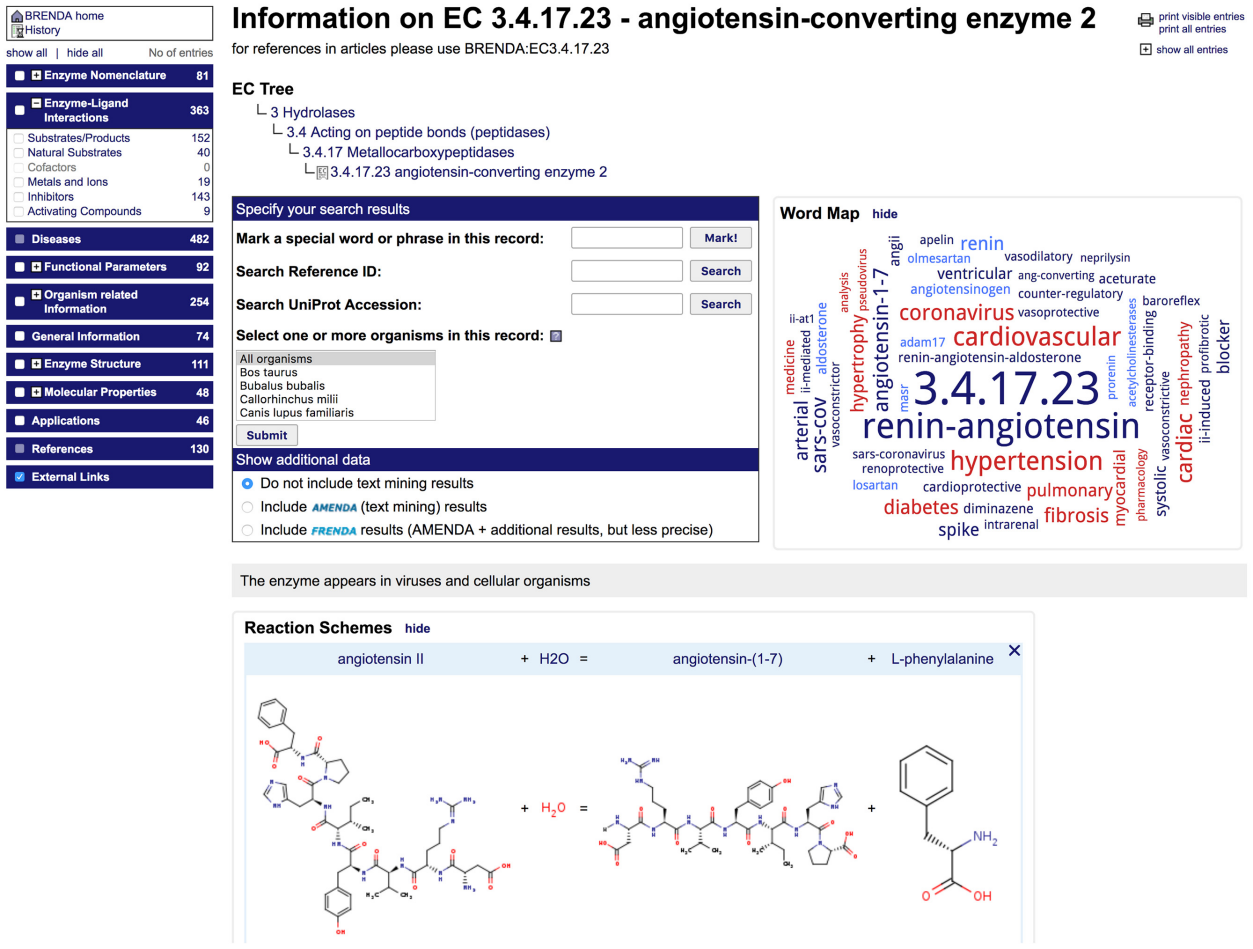
Enzyme summary page for EC 3.4.17.23 providing a quick overview on all aspects of the enzyme. The navigation area on the left side shows the new main categories including the number of entries. The ‘Enzyme-Ligand Interactions’ are expanded to show the included information fields.

When a user visits the Enzyme Summary Page, the first new impression will be the considerably enlarged word map, giving a quick view on all aspects of the enzyme, the reaction scheme of the catalyzed reaction, and the classification of the enzyme within the EC system of the IUBMB (Figure 1).

The navigation box on the left side contains all data fields newly classified in 11 main categories (instead of the former 6): enzyme nomenclature, enzyme-ligand interactions, diseases, functional parameters, organism-related information, general information, enzyme structure, molecular properties, applications, references and external links. Depending on the interests of the user (biochemistry, biotechnology, medicine, pharmacology, microbiology, plant science, systems biology) different information should be displayed on the page to avoid unnecessary scrolling. Therefore, the users can individually select the categories and data fields of interest to be displayed or directly jump to the desired table. The different information fields are collapsed by default and shown on mouseover. The number of entries in the tables are directly displayed. Data fields without any entries are disabled (grayed out).

Above each of the data tables the user can directly switch to the specific search in order to expand or limit the results or compare them with other enzymes.

Additionally, the calculation of an enzyme's expected taxonomic range has been extended by information derived from UniProt. Due to the therefore increased calculation time the expected taxonomic range is now precalculated.

### Localization prediction

The intracellular localization of an enzyme has a crucial effect on its function in organisms. The prediction of transmembrane-helices in enzyme sequences has been a feature in BRENDA for years (TMHMM, (24)). To extend this function the prediction of cellular localizations for eukaryotic enzymes based on N-terminal sequence motifs and other sequence properties using Target-P 1.1 was implemented (25). Possible cellular compartments for the prediction are *secretory pathway, chloroplast, mitochondrion*, etc. Reliability scores ranging from 1 (very reliable) to 5 (not reliable) classify the predictions. These scores indicate the difference between the highest and second highest output scores derived from Target-P 1.1.

The results present 57 627 enzymes to be transported into the *chloroplast*, 10 565 within the highest reliability class, 280,191 into the *mitochondrion*, 38 641 with the highest reliability index, ∼2 million enzymes are predicted to be *secreted*, 861 023 within the highest reliability class, respectively.

The results of the localization prediction will be included into the Enzyme Summary Pages in the upcoming BRENDA release in February 2021.

### New 3D viewer

The previously used viewer for enzyme 3D structures, JSMOL (26), has been replaced by the NGL viewer (27) which is more user-friendly and provides a number of additional features. These attributes comprise a better resolution, the identification of atoms and bonds on mouseover, a color selection for highlighting details of the structure, e.g. the active center, and an easy change between different color schemes, protein and ligand styles. Furthermore, the display of water molecules and ions can be enabled or disabled without having been selected in advance.

### Quality control

New website releases as well as the core databases are constantly and extensively checked by the entire BRENDA team. To further improve the quality of new releases an automatic website check has been developed. The corresponding program offers the possibility to test hundreds of various search combinations of all research options in BRENDA in a short time. In this manner, the current and the previous release as well as different BRENDA webservers can be compared. The web pages are parsed and analyzed to ascertain the availability of the web pages and to find broken links within BRENDA and to external websites. Additionally, the software determines the response time, the number of results of the queries and the presence of important elements, e.g. the word map on the Enzyme Summary Pages. Finally, the program searches for improperly displayed characters. The error and access logs of the various webservers are automatically compared to find PHP errors.

### The revised and extended metabolic pathways

The BRENDA pathway maps initially integrated in 2016 offer an overview on metabolic pathways and biochemical processes. They visualize chemical reactions as well as associated enzyme and ligand information within the metabolism of an organism.

### Map data creation

The creation of the maps as well as extensions and modifications are performed manually with Cytoscape (28), an open source software platform for visualizing complex networks. Currently, the overview map contains 169 metabolic pathways of different sizes. The pathways comprise a total of 11 134 nodes representing 1843 different enzymes, 1953 different metabolites and 2393 reactions.

The complete information in the manually created Cytoscape file is exported as an XGMML file and then imported into a relational database. In an automatic validation process all reaction equations are checked for correctness based on a comparison of all nodes and edges with the respective metabolite, co-metabolite and enzyme information in BRENDA. Before acceptance all errors are corrected and missing sources like identifiers and references are complemented.

**Figure 2.**
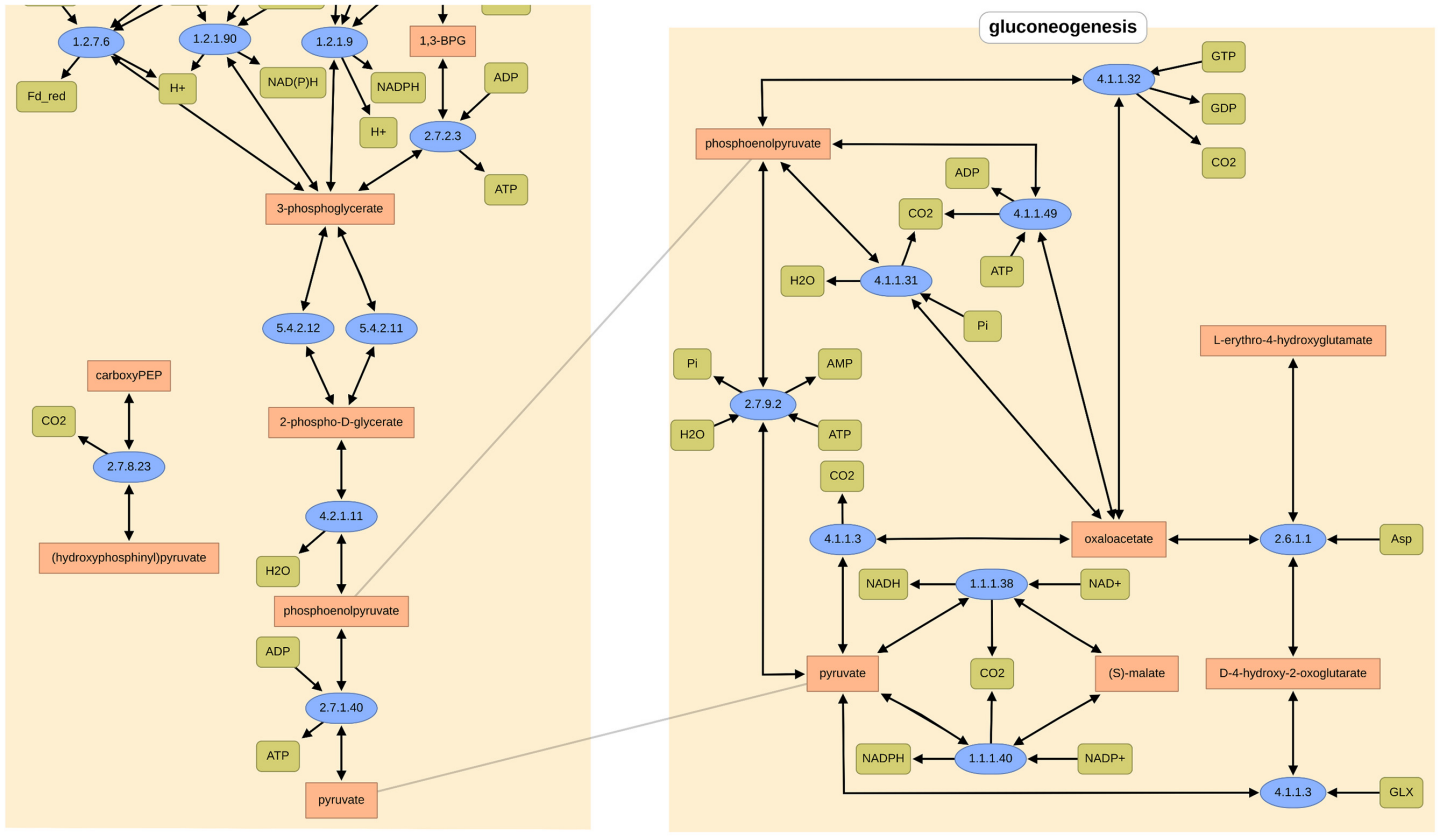
*Interpathway links* in the pathway maps of phosphoenolpyruvate and pyruvate between the glycolysis on the left and the gluconeogenesis on the right.

For the revision in 2019/2020 numerous manually corrections were performed in sources, reactions, metabolites, and enzymes. Furthermore, various pathways, *e.g*. the cellulose degradation or gallate degradation, have been extended or completely reconstructed. Additionally, *interpathway links* were introduced. These are used to show the links between identical metabolites of two pathways and provide a better understanding of the connections between them (Figure 2).

The final XGMML file of the manually plotted pathways is then automatically transformed into an SVG-formatted file for the online visualization.

### Pathway visualization

**Figure 3.**
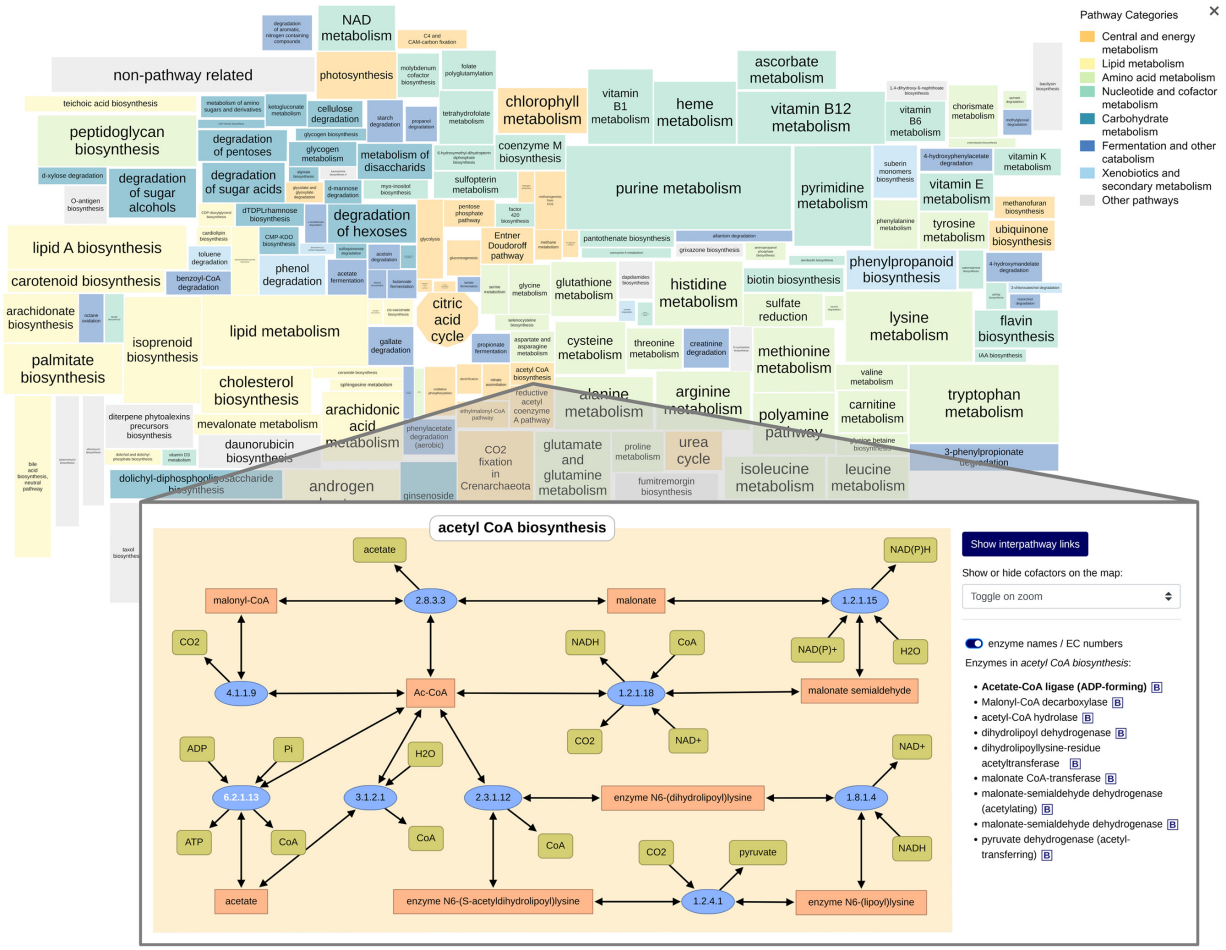
Pathway categories in the overview map and a detailed pathway view. The upper part of this figure shows the interactive overview map with pathway backgrounds colored differently with respect to metabolic roles and categorized as *central and energy metabolism*, *lipid metabolism*, *amino acid metabolism*, *nucleotide and cofactor metabolism*, *carbohydrate metabolism*, *fermentation and other catabolism* as well as *xenobiotics and secondary metabolism*. The lower part of this figure illustrates the detailed pathway view of the acetyl CoA biosynthesis with the highlighted enzyme node 6.2.1.13-acetate-CoA ligase and the enzyme list on the right side.

The online visualization procedure has been completely rewritten. The former separate pathway maps have been replaced by an interactive global map (Figure 3) including all pathways with a zoom function comprising different levels of details depending on the zoom factor, thereby increasing the visualization performance by orders of magnitude. The user can switch between the overview map and a detailed pathway view with or without co-metabolites by zooming in and out.

Each pathway can be selected and centered. The pathway backgrounds are colored differently with respect to metabolic roles and categorized as *central and energy metabolism*, *lipid metabolism*, *amino acid metabolism*, *nucleotide and cofactor metabolism*, *carbohydrate metabolism*, *fermentation* and *other catabolism* as well as *xenobiotics and secondary metabolism*. Shown on the right side is an enzyme list of the selected pathway with direct links to the Enzyme Summary Pages (Figure 3). The user can decide whether cofactors should be displayed always, never or depending on the zoom level. On the left side, an interaction panel with the sections ‘Search & Highlight’, ’Plot Your Dataset’ and ‘Download Map as SVG’ has been implemented.

### Pathway query options

**Figure 4.**
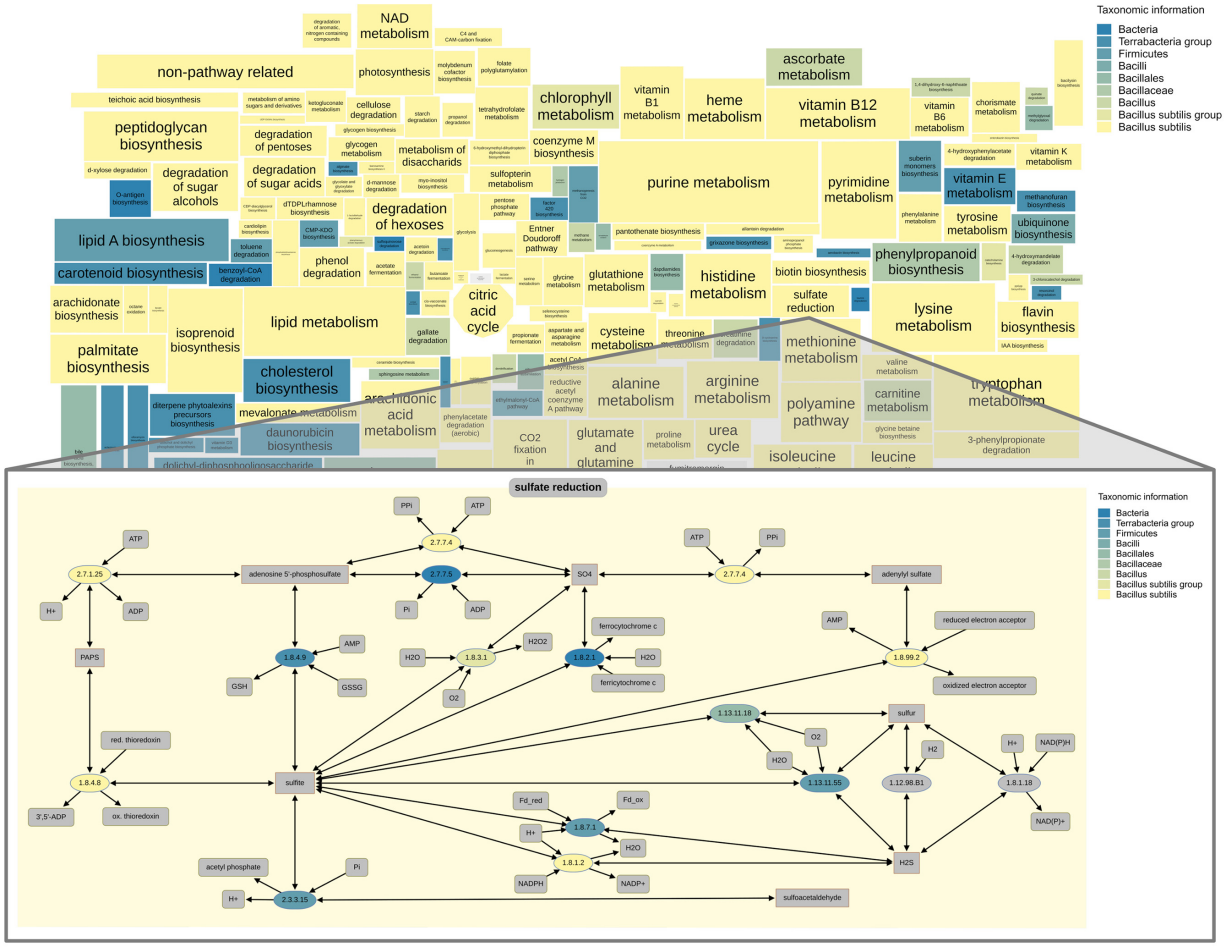
The overview map shows the pathway coverage of *Bacillus subtilis* including all taxonomically related ranks up to the domain Bacteria whereas the example map shows the enzyme coverage for the sulfate reduction.

An intuitive and quick search for pathways, metabolites, and enzymes within the maps is possible in the ‘Search & Highlight’ section. Furthermore, the metabolic capacity of an organism or taxon can be visualized. The corresponding sources for this search like manually verified BRENDA data, BRENDA’s text mining data, SwissProt, and TrEMBL can be selected below the organism/taxon input field. By using ‘visualize taxonomic information’ pathways of closely related organisms are indicated. The metabolic repertoire for *Bacillus subtilis* including all taxonomically related ranks up to the domain Bacteria is exemplified in Figure 4. The overview map shows the coverage in all BRENDA maps whereas the example map shows the coverage for the sulfate reduction. Here, the enzyme nodes and pathway backgrounds are highlighted according to the different taxa from the organism of interest in light yellow to the bacterial level in blue.

### Embedding experimental data in pathways

For the first time the new pathway map allows users to visualize their own experimental omics-datasets using the ‘Plot Your Dataset’ functionalities. Therefore, multi-omics data and flux distributions can be visualized and analyzed in their metabolic context. The data plotting uses identifiers (e.g. the EC number or metabolites) that connect each data point with a certain node on the metabolic map.

**Figure 5.**
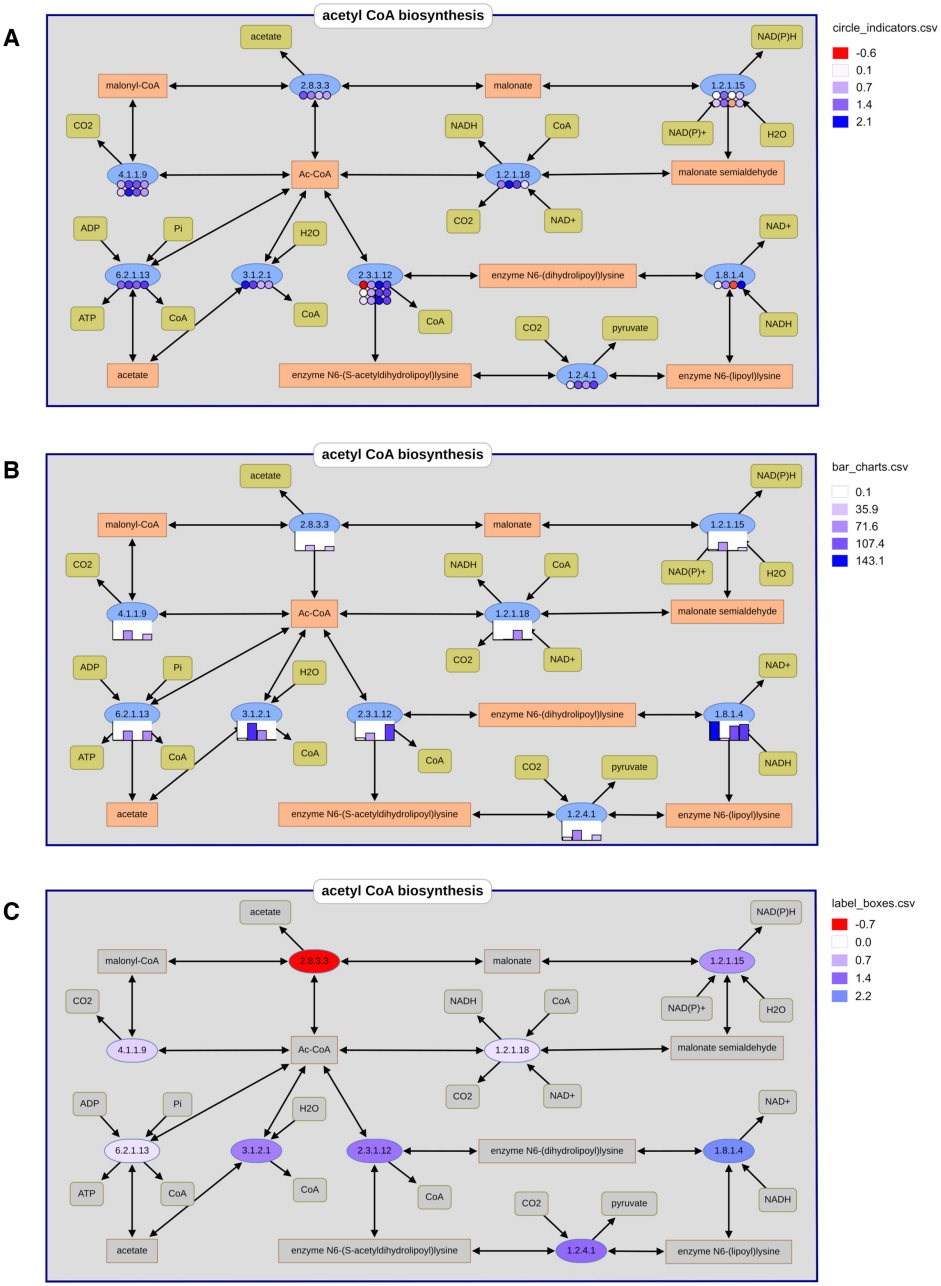
An example of the three different plot types for experimental user data in the acetyl-CoA biosynthesis: (**A**) circle indicators, recommended for transcriptome and proteome data, since duplicate identifiers can be visualized, e.g. when more than one gene encodes for the same enzyme. (**B**) bar charts, recommended for metabolome data, since they only support positive data values and only one occurrence of each identifier. (**C**) label boxes, recommended for transcriptome data where two conditions are given as log2fold-changes and one node represents one dataset. The figure is based on exemplary data to illustrate the visualization.

The new BRENDA pathway map offers three different plot types for experimental values: (A) circle indicators, a recommended plot type for transcriptome and proteome data, since visualizing of duplicate identifiers is possible. This may occur when the organism has more than one gene that encodes for the same enzyme. (B) bar charts, which are recommended for metabolome data, since they only support positive data values and only one occurrence of each identifier. (C) label boxes, where the node itself is colored based on the data value. This is the recommended plot type, when one dataset shall be visualized, e.g. transcriptome data where two conditions are given as log_2_-fold changes. An example is given in Figure 5. The figure is based on exemplary data to illustrate the visualization.

### Download of the pathway map

In the ’Download’ section, the user can download parts of the BRENDA maps. Additionally, all modifications to the map, including graphs and visualizations are also visible.

### EnzymeDetector

A correct genome annotation is essential to understand cellular functions and the metabolism of an organism. An important aspect is the prediction of enzymatic functions to identify biochemical reactions in a metabolic pathway.

BRENDA’s tool EnzymeDetector (https://ed.brenda-enzymes.org/) provides an integrative overview of enzymatic function annotations obtained from major annotation hosts supplemented with prediction results for ∼8300 organisms and 18 million genes. As part of a fundamental revision of the website new features were implemented, links to further resources were added and all data was updated.

### Data increase and prediction sources

Since its last release in 2017 the number of enzyme function predictions for bacterial genomes and archaeal genomes has increased by more than 20 million. In addition, the data was extended by 1230 eukaryotic genomes and more than 15 million predictions for eukaryotes. The enzymatic function predictions are obtained from BRENDA, AMENDA, KEGG, NCBI’s RefSeq (29), UniProt, a sequence similarity analysis via BLAST (30), and sequence pattern searches via BREPS (31). For bacteria and archaea the annotation data from PATRIC (32) are also integrated. Since the amount of data and annotations differ between these popular prediction hosts, the easy and simultaneous access to all sources via the EnzymeDetector is a significant advantage.

### The optimized annotation overview

The organism search on the entry page leads to the corresponding annotation overview and further information to the organism like new external links to the NCBI taxonomy, BRENDA and BacDive. Based on the agreement of the different sources, a confidence score is calculated for each prediction in order to identify annotation errors and assess the data reliability. The confidence score results from the sum of the weighted organism-domain-specific reliability of a source, which is determined by the overall agreement between its predictions and the manual EC-classification by the BRENDA team.

**Figure 6.**
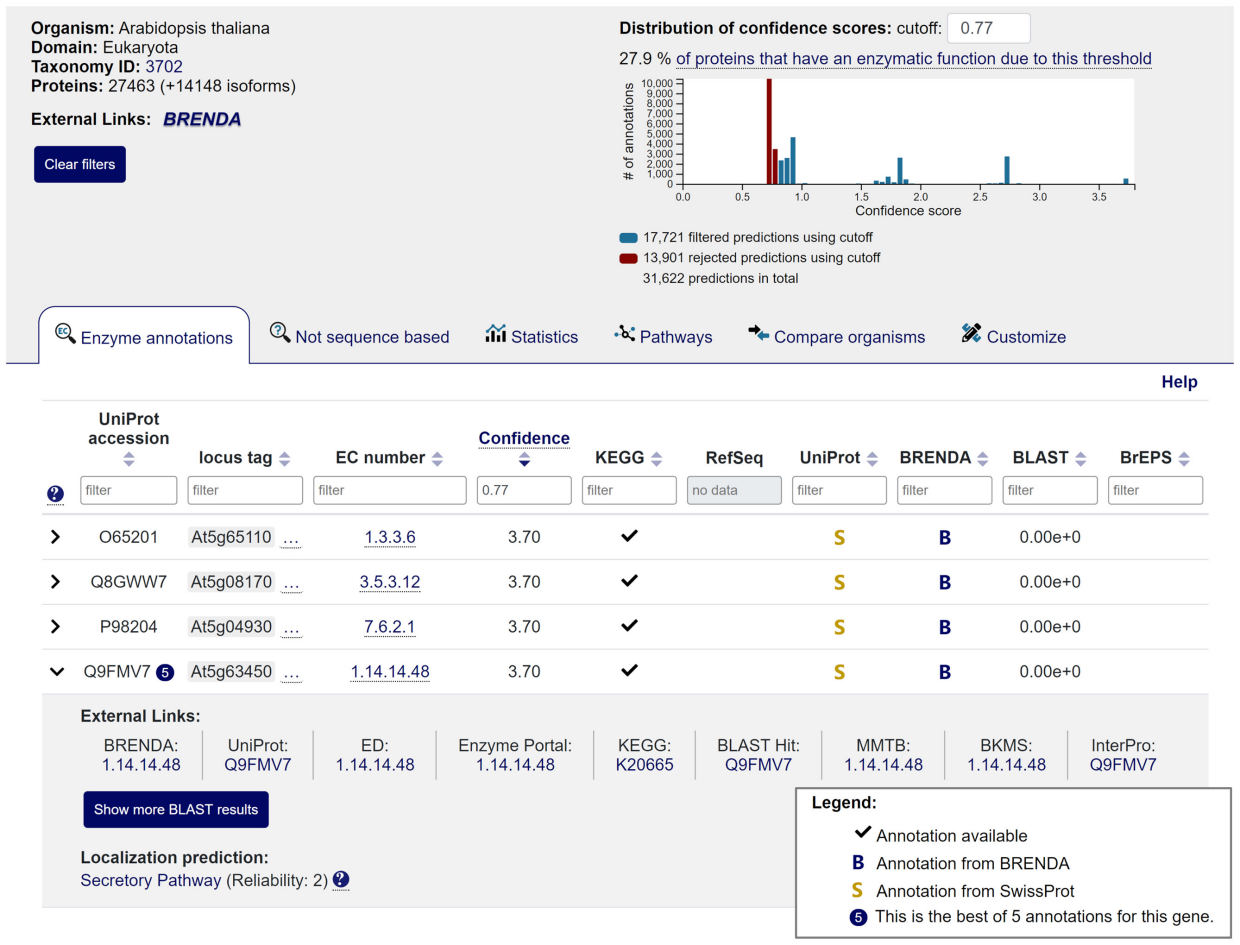
The EnzymeDetector annotation overview of *Arabidopsis thaliana* with the histogram representing the distribution of the confidence scores and the circular note behind the UniProt Sequence ID Q9FMV7 in the fourth line of the table pointing to five different annotations for this sequence.

A histogram with the distribution of the confidence scores of all predictions has been newly integrated to facilitate the definition of the cutoff (Figure 6). By using this cutoff unreliable predictions can be filtered out and the percentage of proteins that have an enzymatic function due to this threshold can be computed.

Several annotations for a sequence can now be recognized more easily by the number in the circle behind the given UniProt Sequence ID. There are also additional external links for each prediction to the EBI Enzyme Portal (33), KEGG, PATRIC, BrEPS, the best BLAST hit, BKMS-react, InterPro (34) and MMTB. The Metano Modeling Toolbox (MMTB) is an online tool based on the data of BKMS-react that assists metabolic modeling by providing an interface to the Metano software (35). Via ‘Show more BLAST results’ it is possible to display the 10 best BLAST hits for a sequence. The data of BRENDA’s Localization Prediction for eukaryotic sequences are also integrated.

### Further improved annotation features

**Figure 7.**
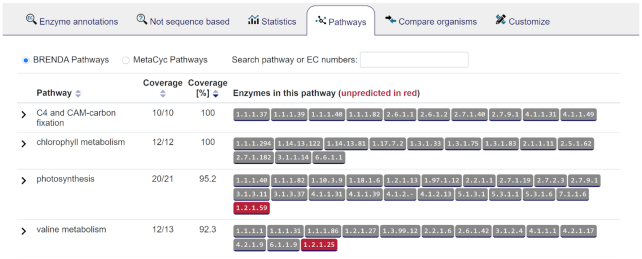
The ’Pathways’ section of *Arabidopsis thaliana* with predicted EC numbers in gray and missing EC numbers in red (page 2).

Not-sequence-based annotations of BRENDA and BRENDA’s text mining method AMENDA are now available in a new tab with external links, reliability classes and a list of references for each annotation. The section ‘Pathways’ is newly classified in BRENDA and MetaCyc Pathways. Predicted EC numbers are displayed in gray and missing EC numbers in red (Figure 7). In the section ‘Customize’ the user can adjust the reliability of the sources and download the resulting annotation table in JSON or CSV format.
